# Role of Ambient Hydrogen in HiPIMS-ITO Film during Annealing Process in a Large Temperature Range

**DOI:** 10.3390/nano12121995

**Published:** 2022-06-10

**Authors:** Ming-Jie Zhao, Jin-Fa Zhang, Jie Huang, Zuo-Zhu Chen, An Xie, Wan-Yu Wu, Chien-Jung Huang, Dong-Sing Wuu, Shui-Yang Lien, Wen-Zhang Zhu

**Affiliations:** 1 School of Opto-Electronic and Communication Engineering, Xiamen University of Technology, Xiamen 361024, China; 2015000077@xmut.edu.cn (M.-J.Z.); jfzhang2019@stu.xmut.edu.cn (J.-F.Z.); huangjie@stu.xmut.edu.cn (J.H.); 2022031141@s.xmut.edu.cn (Z.-Z.C.); wzzhu@xmut.edu.cn (W.-Z.Z.); 2 Fujian Key Laboratory of Optoelectronic Technology and Devices, Xiamen University of Technology, Xiamen 361024, China; 3 School of Materials Science and Engineering, Xiamen University of Technology, Xiamen 361024, China; anxie@xmut.edu.cn; 4 Department of Materials Science and Engineering, Da-Yeh University, Dacun, Changhua 51591, Taiwan; wywu@mail.dyu.edu.tw; 5 Department of Applied Physics, National University of Kaohsiung, Kaohsiung University Rd., Kaohsiung 81148, Taiwan; chien@nuk.edu.tw; 6 Department of Applied Materials and Optoelectronic Engineering, National Chi Nan University, Nantou 54561, Taiwan; dsw@ncnu.edu.tw

**Keywords:** indium tin oxide (ITO), HiPIMS, rapid thermal annealing (RTA), hydrogen-containing forming gas, hydrogen doping

## Abstract

Indium tin oxide (ITO) thin films were prepared by high power impulse magnetron sputtering (HiPIMS) and annealed in hydrogen-containing forming gas to reduce the film resistivity. The film resistivity reduces by nearly an order of magnitude from 5.6 × 10^−3^ Ω·cm for the as-deposited film to the lowest value of 6.7 × 10^−4^ Ω·cm after annealed at 700 °C for 40 min. The role of hydrogen (H) in changing the film properties was explored and discussed in a large temperature range (300–800 °C). When annealed at a low temperature of 300–500 °C, the incorporated H atoms occupied the oxygen sites (H_o_), acting as shallow donors that contribute to the increase of carrier concentration, leading to the decrease of film resistivity. When annealed at an intermediate temperature of 500–700 °C, the H_o_ defects are thermally unstable and decay upon annealing, leading to the reduction of carrier concentration. However, the film resistivity keeps decreasing due to the increase in carrier mobility. Meanwhile, some locally distributed metallic clusters formed due to the reduction effect of H_2_. When annealed at a high temperature of 700–800 °C, the metal oxide film is severely reduced and transforms to gaseous metal hydride, leading to the dramatic reduction of film thickness and carrier mobility at 750 °C and vanish of the film at 800 °C.

## 1. Introduction

Transparent conductors characterized by high conductivity and high transparency in the visible light spectrum have been widely used in many optoelectronic applications, such as photovoltaic cells, flat panel displays, light-emitting diodes and smart windows [1,2,3,4]. Indium tin oxide (ITO) is an excellent transparent conductor. Although it has been investigated for decades, it is still regarded as the most typical and important transparent conductor due to its high optical transmission, low electrical resistivity, high stability against harsh environments and easy processing properties. ITO film is usually prepared by DC or RF magnetron sputtering, which is capable of depositing uniform, large-area ITO film with a high deposition rate [5,6,7]. However, the film quality is somewhat inferior, especially when deposited at low temperatures. Persistent efforts have been devoted to improving film quality, aiming at increasing the optical and electrical properties of the film with various approaches, including doping, process parameter optimization, post-deposition thermal or chemical treatment and deposition method innovation [8,9,10,11,12]. 

Recently, high power impulse magnetron sputtering (HiPIMS) is emerging as a method for deposition of metal oxide films, including ITO films [13,14,15,16,17]. The high power impulses can generate high-density plasma containing highly excited/ionized sputtered species and reactive agents (oxygen radicals), favoring the synthesis of compounds. In addition, the film conformality can be enhanced thanks to the highly energetic plasma species, which is applicable for a 3D substrate such as devices with a high aspect ratio. Adding hydrogen gas to the sputter gas during film deposition or to the ambient during the annealing process and hydrogen plasma treatment has been proved to be effective approaches to reducing the film resistivity [18,19,20,21]. Moreover, hydrogen incorporation in oxide film by either annealing in an H_2_ ambient at elevated temperature or hydrogen plasma treatment has been reported in the literature. However, these approaches have not been applied to ITO films prepared by the HiPIMS method. Furthermore, the role of hydrogen in influencing the properties of ITO film is still controversial so far. Some researchers stated that incorporated H exists as interstitial (H_i_) or occupies the oxygen sites (H_o_), both of which act as a shallow donor in metal oxide films, leading to the increase of carrier concentration and the decrease of film resistivity [22,23]. Some researchers argued that neither the interstitial hydrogen nor the substitutional hydrogen was likely the origin of incremental free carrier concentration in the film. Rather, oxygen vacancy defects created by H_2_ reduction contribute to incremental free carriers [24,25,26,27,28,29,30]. The experimental conditions are not exactly the same as the existing reports. For instance, the investigated temperature ranges (not higher than 500 °C in most of the research in the literature), the hydrogen source (hydrogen plasma or hydrogen-containing atmosphere with various hydrogen proportions) and the introduction stage (during the film deposition process or annealing process) varied from each other. To clarify the role of hydrogen, investigations should be taken in a larger temperature range with consistent experiment setups throughout the temperature range. Nevertheless, this is still in lack so far. Furthermore, most research on the hydrogen annealing effects of ITO films focused on the analysis of the variation of oxygen sub-stoichiometry state of the film influenced by the annealing process. However, less attention has been paid to the possible material exchange between the film and the annealing ambient, such as the adsorption/desorption of hydrogen, hydroxyl groups, water, etc.

Previously, ITO films were prepared through the HiPIMS method by our group [31,32]. In this work, the films were annealed in hydrogen-containing forming gas to reduce film resistivity. The role of hydrogen in influencing the film properties is explored and discussed at a large temperature of 300–800 °C.

## 2. Materials and Methods

ITO thin films were deposited on a quartz glass substrate and silicon substrate by an in-line sputtering system (Ljuhv, SP-122I, Zhubei City, Taiwan) using a high-purity (99.99%) ceramic target consisting of 90 wt.% In_2_O_3_ and 10 wt.% SnO_2_ (supplied by Heqi Target Material Technology, Zhangzhou, China). The target has a dimension of 30.7 cm (length) × 12.5 cm (width) × 0.6 cm (thickness). The sputtering system was described in detail in References [31,32]. The film deposition parameters (working pressure and sputtering power) have been optimized and reported in previously published papers. The base pressure of the process chamber was lower than 6.7 × 10^−^^5^ Pa. The high-purity (99.999%) Ar with a flow rate of 40 sccm was used as the plasma gas. The sputtering pressure was kept at 8.0 Pa during the film deposition process. The average power, duty cycle, pulse frequency and pulse length were set at 500 W, 10%, 1000 Hz and 100 us, respectively. The substrate was not heated during the film deposition process. The size of the deposited samples was 5 cm×5 cm. The inhomogeneity of film thickness was evaluated by a nine-point method. The film thicknesses (*d*) at nine points distributed in a substrate of 10 cm × 10 cm were measured by a step profiler. The inhomogeneity determined by (*d*_max_ − *d*_min_)/(*d*_max_ + *d*_min_) is within 2%. The deposition time was 10 min. The deposition rate was 8 nm/min. The deposition parameters for the HIPIMS process are summarized in Table 1.

The obtained ITO film was annealed in a forming gas containing 5% hydrogen (H_2_) and 95% nitrogen (N_2_) at 300–800 °C for 40 min using a rapid thermal annealing system (Eaststar Labs, RTP-300, Beijing, China). The forming gas with a total flow rate of 1 L/min was introduced into the furnace during the whole process. The annealing process consisted of three stages: (1) the temperature rose from room temperature to 200 °C in 20 s, then held for 20 s; (2) the temperature rose from 200 °C to the aiming temperature (300, 400, 500, 600, 700, 750 and 800 °C for each specimen, respectively), then held for 40 min; (3) The temperature fell from the aiming temperature to room temperature in about 500 s. Parameters for the annealing process of ITO film are summarized in Table 2.

The film thickness (*d*) was measured from the field emission scanning electron microscope (FESEM, sigma 500, Zeiss, Oberkochen, Germany) and captured images of a cross-section of the ITO/quartz specimens before and after the annealing process. The infrared (IR) absorption spectra of the films were measured with a Fourier transform infrared (FTIR, Bruker Vertex80v, Ettlingen, Germany) spectrometer. The depth distribution of elements in the film was analyzed using time-of-flight secondary ion mass spectrometry (TOF-SIMS, ION-TOF, TOF-SIMS V, Muenster, Germany). The crystal structure of the film was analyzed by grazing X-ray diffraction (GIXRD, Rigaku, TTRAXIII, Ibaraki, Japan) spectra using Cu-Kα radiation (*λ* = 0.154 nm) as the X-ray source. The X-ray incidents with an angle of 1°. The surface morphology of the films was observed by FESEM and atomic force microscopy (AFM, Park, XE7, Suwon, Korea). The roughness (*R*_q_) of the film surface was obtained by statistical analysis of the AFM data. The cross-section of the film was prepared by a focused ion beam (FIB, FEI Helios, Hillsboro, OR, USA) and observed by a high-resolution transmission electron microscope (TEM, FEI Talos F200X, Hillsboro, OR, USA). The electrical properties were measured by a four-point probe station (Ossila, T2001A3, Sheffield, UK) and a Hall effect system (Hall, Ecopia, HMS5000, Anyang, Korea). The transmittance of the specimens was measured by a spectrometer (PerkinElmer Lambda 850, Waltham, MA, USA). The film transmittance (*T*) was obtained by deducting the transmittance of bare quartz glass substrate from that of ITO/quartz specimens. The film characterization was performed on samples on quartz glass substrate except for the analyses of SIMS and XPS, which were performed on samples on a silicon substrate.

## 3. Results and Discussion

**Figure 1:**
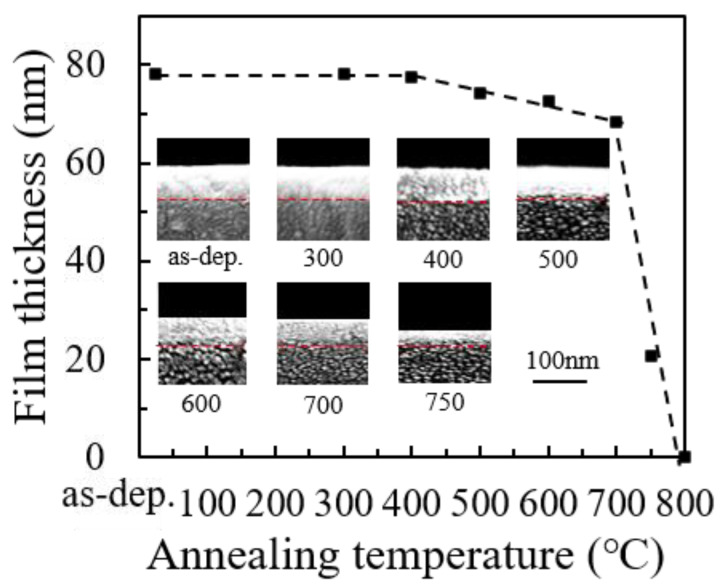
The variation of thickness of ITO film with annealing temperature. The inset shows the corresponding FESEM images of the specimens for the measurement of film thickness.

Figure 1 shows the variation of film thickness with annealing temperature. The inset shows the FESEM images of a cross-section of the specimens for the measurement of film thickness. The graphs in Figure 1 present seven different specimens separated from the deposited sample. The discrepancy of the original film thickness should be within 2%. The thickness of the as-deposited film was 78.2 nm. The film thickness is almost unchanged after annealing at 300 and 400 °C. Then, it modestly decreases after annealed at 500–700 °C, possibly due to the partial reduction of the oxide film by H_2_. Finally, it dramatically decreased after annealed at 750 °C and totally vanished after annealed at 800 °C due to severe reduction and vaporization of the film. The chemical reaction of the film material and the ambient gas will be discussed later.

Generally, the Sn content and O content in the film are important factors that influence the number of donor defects such as Sn substitutions and oxygen vacancies, thus leading to the variation of carrier concentration in the film. Therefore, most research elaborated on the oxygen sub-stoichiometry states of the ITO film. In previous works, the relative Sn doping efficiency (reflected by the areal ratio of Sn^4+^/(Sn^4+^ + Sn^2+^) in the Sn 3d XPS peaks) and the relative oxygen vacancy concentration (reflected by the areal ratio of O_V_/(O_V_ + O_L_) in the O 1s XPS peak) in the HiPIMS-ITO film have been investigated as functions of working pressure and average power during the film deposition process [31,32]. The same analyses have been performed on the samples in this work. The results are listed in Table 3 and compared with previous works of our group. The Sn doping efficiency first decreases with annealing temperature at 25–500 °C, then increases with annealing temperature at 500–750 °C. The variation of Sn doping efficiency is in the range of 69.8–75.0% when the annealing temperature varies from 25 to 800 °C. In contrast, in our previous works, the variations of Sn doping efficiency are in the ranges of 32.6–69.6% and 65.6–82.2%, as the average power varies in the range of 200–600 W and the working pressure varies in the range of 5.3–10.0 Pa, respectively. The oxygen vacancy concentration is shown in Table 3 first increases with annealing temperature at 25–500 °C, then decreases with annealing temperature at 500–750 °C. The variation of oxygen vacancy concentration is in the range of 42.0–47.6% when the annealing temperature varies in the range of 25–800 °C. In contrast, in our previous works, the variations of oxygen vacancy concentrations are in the ranges of 39.0–48.1% and 42.1–53.5%, as the average power varies in the range of 200–600 W and the working pressure varies in the range of 5.3–10.0 Pa, respectively. Therefore, the variations of Sn doping efficiency and oxygen vacancy concentration with annealing temperature in this work are much smaller than with average power and working pressure in our previous work. However, the annealing treatment leads to a much larger variation in the carrier concentration (Δ*N*_c_ = 9.3 × 10^20^ cm^−3^, see the results of Hall effect measurement) compared to the smaller Δ*N*_c_ = 2.2 × 10^20^ cm^−3^ and Δ*N*_c_ = 5.6 × 10^20^ cm^−3^ by varying average power and working pressure. Although the variations in Sn doping efficiency and oxygen vacancy defects would influence the carrier concentration in the film, they may not be the dominant factors. Therefore, we infer that the variation of carrier concentration with annealing temperature should be primarily attributed to the possible material exchange between the film and the annealing ambient rather than the Sn doping efficiency or the slight oxygen sub-stoichiometry state. Therefore, the analysis of the hydrogen-related defects is of main concern in this work.

**Figure 2:**
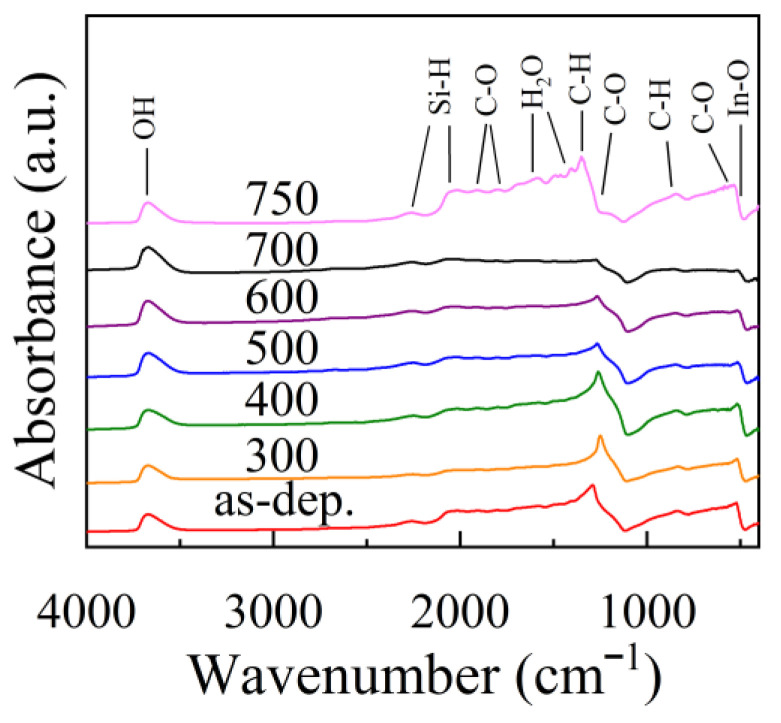
The FTIR spectra for ITO films deposited at different temperatures.

The hydrogen centers in ITO film were studied by FTIR absorption spectra, as shown in Figure 2. The assignment of the infrared absorption peaks in the FTIR spectra is summarized in Table 4. The peak located at 3680 cm^−1^ origins from the vibration of OH species [33]. The peak at 520 cm^−1^ is attributed to the vibration of the In-O bond [34,35]. The peak intensity slightly decreases with increasing annealing temperature at 300–700 °C, possibly due to the removal of weak In-O bonds by annealing. A broad band in the range of 1291–2260 cm^−1^ was observed for the as-deposited film, which possibly originated from the C, H and H_2_O-related impurities in the film. It becomes weaker with increasing annealing temperature at 300–700 °C due to the removal of these impurities by annealing. It has been reported in the case of AZO films deposited by reactive HiPIMS stored in ambient air contain hydroxyl groups in the bulk of the film, and these groups are released after subsequent annealing in reducing atmosphere [36]. The film resistivity significantly decreases after this release. In this case, a similar phenomenon may occur in HiPIMS-ITO films. The water content in the as-deposited film is quite high and drops after annealing. The release of the adsorbed water may contribute to the decrease of film resistivity after annealing. However, the intensity of the broadband becomes stronger and with featured peaks superimposed on it after annealing at 750 °C. The featured peaks should be assigned to the stretching vibration of Si-H, C-O, H_2_O and O-H bonds [37,38,39,40,41,42,43,44]. It is speculated that these featured peaks originated from the adsorbent on the film surface, which is porous and rough, as observed by FESEM.

**Figure 3:**
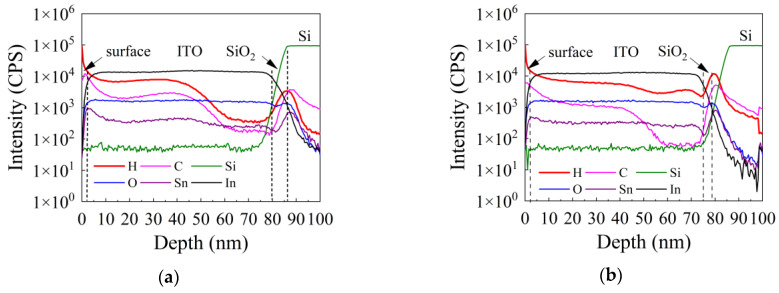
The SIMS depth profile for the (**a**) as-deposited ITO film and (**b**) 500 °C-annealed ITO film, respectively.

Figure 3a,b shows the SIMS depth profile for the as-deposited and 500 °C-annealed ITO films, respectively. The intensity (in the unit of counts per second, CPS) in the SIMS spectra reflects the relative concentration of the certain element. The variation of the concentration of certain elements along the depth direction can be observed in the spectra. For the as-deposited ITO film, the intensity of H and C at the near-surface of the film is almost 1.5 orders higher than that in the bulk of the film, suggesting that the H and C in the as-deposited film are probably uptake from the ambient due to ambient exposure of the specimens. The Sn element seems to be unevenly distributed along the depth of the film. For the 500 °C-annealed ITO film, the distribution of C is similar to that of the as-deposited film. However, the C content in the annealed film is smaller than that in the as-deposited film both at the near film surface and in the bulk of the film. The integral SIMS intensity of C from 0 to 80 nm for both the as-deposited and annealed film was calculated. The result shows that the integral intensity of C decreases from 1.7 × 10^5^ CPS·nm/s to 9.1 × 10^4^ CPS·nm/s, suggesting the C related impurity was eliminated after annealing. This is in accordance with the analysis of FTIR results. However, the intensity of H in the bulk of the film increases to the level that is close to the near-surface of the film. Meanwhile, the amount of hydrogen tends to have lowered around 40 nm of etching. This result suggests that hydrogen redistribution and hydrogen adsorption from the ambient may occur during the annealing process. To verify the hydrogen adsorption, the integral SIMS intensity of H from 0–80 nm for both the as-deposited and annealed film was calculated. The integral intensity of H increases from 4.3 × 10^5^ CPS·nm/s to 5.0 × 10^5^ CPS·nm/s, indicating that more hydrogen atoms were incorporated into the film. In addition, the Sn element exhibits a more even distribution along the direction perpendicular to the substrate. However, the comparison between the concentrations of different elements cannot be made since the spectra do not give the absolute intensity of the corresponding elements. Therefore, the hydrogen concentrations in the SIMS spectra are not necessarily high since the relative intensity does not give the absolute concentrations.

**Figure 4:**
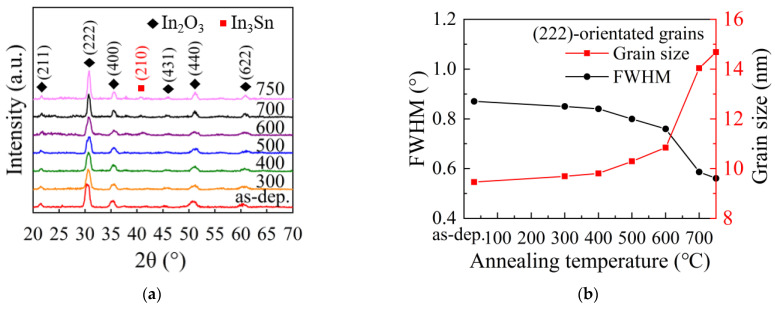
(**a**) XRD patterns of the ITO films annealed at different temperatures. (**b**) The variation of full width at half maximum (FWHM, *β*) of the (2 2 2) peak and corresponding grain size with annealing temperature.

Figure 4a shows the XRD patterns of ITO films annealed at different temperatures. The diffraction peaks for the as-deposited film are all identified to be belonging to the diffraction by (2 1 1), (2 2 2), (4 0 0), (4 3 1), (4 4 0) and (6 2 2) planes of cubic bixbyite In_2_O_3_ lattice (JCPDS Card No. 06-0416). The multiple-peak feature of the pattern reveals the poly-crystal nature of the film with (2 2 2)-planes as the preferentially orientated planes. The cubic bixbyite crystal phase is stable after annealing at 300–500 °C. The full width at half maximum (FWHM, *β*) of the (2 2 2) peak decreases with increasing annealing temperature, as shown in Figure 4b, indicating that the grains grew larger after annealed. The grain size (*D*) was calculated according to Scherrer’s formula [45]:

(1) $$D=\frac{0.94\lambda }{\beta \cos\theta }$$

where λ is the wavelength of the incident X-ray (0.154 nm), and *θ* is the diffraction position. The grain size increases with annealing temperature, as shown in Figure 4b. Noticeably, an emerging peak located at 41.38° was observed when the annealing temperature increased to 600–750 °C, which is assigned to the diffraction by In_3_Sn alloy (JCPDS Card No. 07-0345). The existence of the In_3_Sn phase suggests that the oxide films are partially reduced to the alloy phase after being annealed at such a high temperature. Similarly, In/Sn clusters have been observed in ITO film after H_2_ plasma treatment and annealing treatment in H_2_ containing atmosphere reported by other groups [19,20].

**Figure 5:**
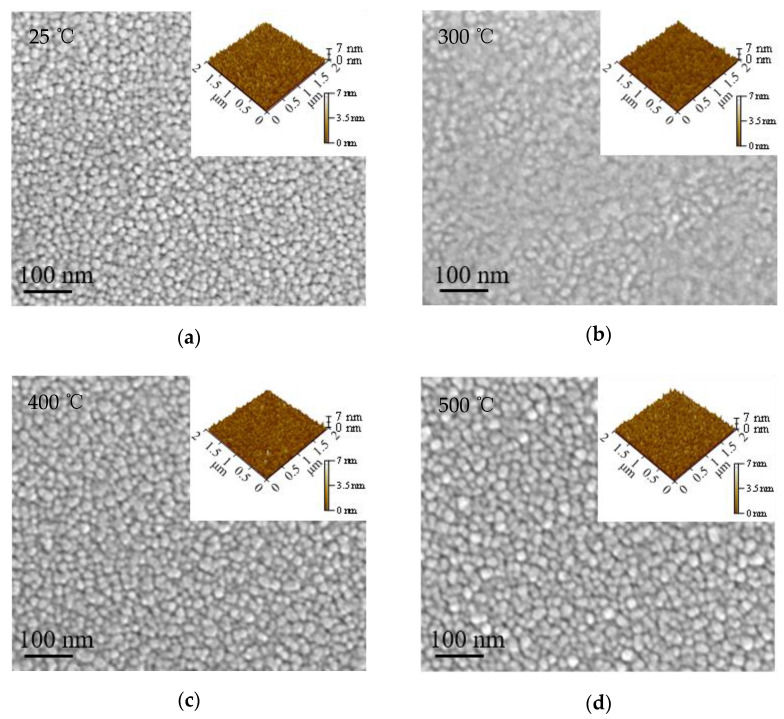

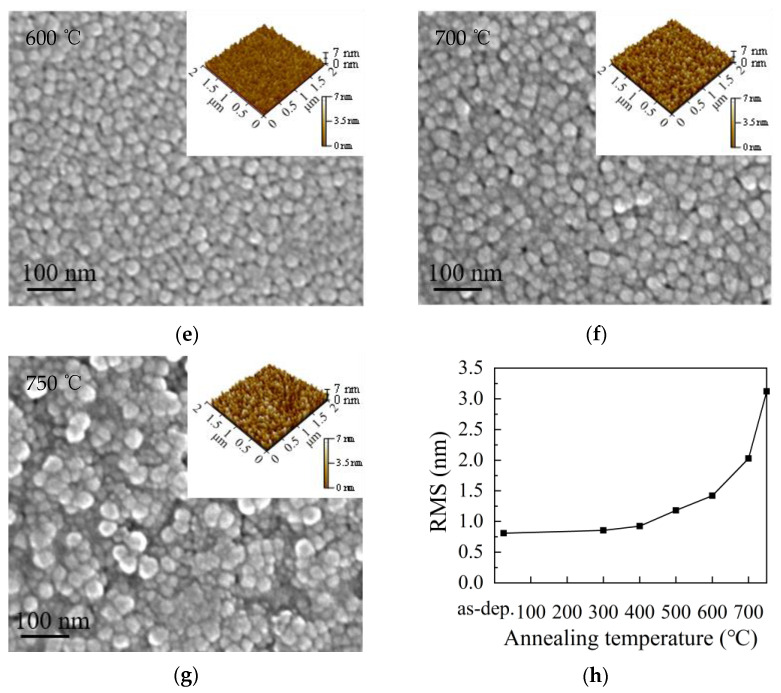
(**a**–**g**) The SEM images for the as-deposited and annealed ITO films (the corresponding AFM images were embedded at the top right corner of the SEM images). (**h**) The variation of film surface roughness with annealing temperature.

Figure 5a–g shows the SEM images for the ITO films. The corresponding AFM images were embedded at the top right corner of the SEM images. The surface roughness of the film is plotted as a function of annealing temperature in Figure 5h. The surface of the as-deposited film consists of uniform grains with clear boundaries. However, the grain boundaries become blurred after annealing at 300 °C. Then, they become clear again when the annealing temperature increases to 400 and 500 °C. The blurred grain boundaries seem to be on a transition stage of molten-to-recrystallization, which exhibits a surface morphology with seemingly amorphous characteristics. The grain size seems to slightly increase as the annealing temperature increases from room temperature to 500 °C. However, coarse grains were observed when the annealing temperature increased to higher than 600 °C. Voids started to appear at 700 °C. Suspected clusters were observed after annealing at 750 °C. The emergence of coarse grains, voids and clusters are likely to be caused by violent structural evolution such as precipitation of metal particles and escape of gaseous materials. As a result, the surface roughness generally increases with annealing temperature, as shown in Figure 5h. The porous and rough film surface after being annealed at 750 °C is more likely to accommodate adventitious contaminants, which is in accordance with the analysis of the FTIR spectra.

**Figure 6:**
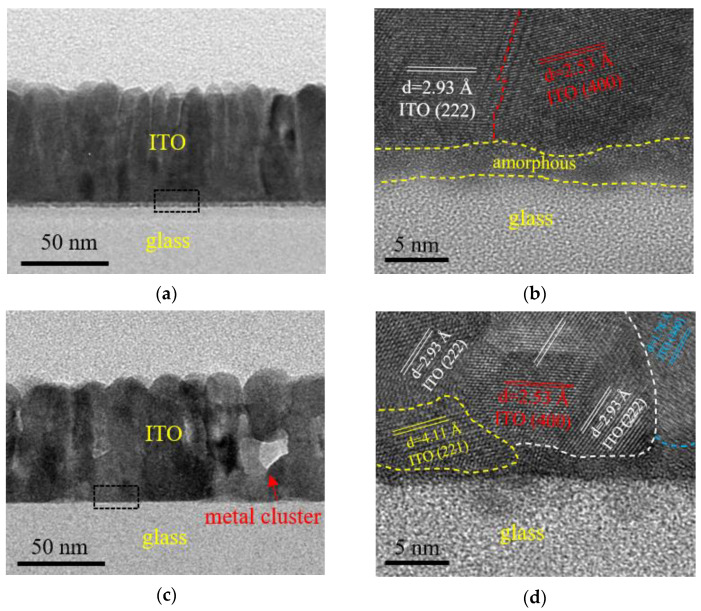
The TEM images for (**a**,**b**) as-deposited and (**c**,**d**) 500 °C-annealed ITO film. The magnification of the images in (**a**,**c**) and (**b**,**d**) is 58 K and 820 K, respectively.

Figure 6a,c shows the TEM images with a magnification of 58 K of as-deposited and 500 °C-annealed ITO films, respectively. Both films exhibit columnar crystals. However, the columnar crystals grew larger after annealing. In addition, a thin amorphous layer was observed at the film/substrate interface for the as-deposited specimen, whereas it disappeared after annealed, suggesting that the crystallinity of the film increases after annealing. Some small lighter-colored regions were observed in the TEM images with a magnification of 58 K (Figure 6c). Since In_3_Sn metallic phase was observed in the XRD patterns for ITO films deposited at ≥600 °C, the lighter-colored region is suspected to be the metallic clusters. Although the XRD peak corresponding to In_3_Sn metallic phase was not observed for the film annealed at 500 °C, it is possible that the proportion of the metallic phase is too small to cause distinct diffraction that can be observed by XRD. Therefore, the observations of TEM and XRD are in accordance with each other. Figure 6b,d show the high-resolution TEM images with a magnification of 820 K, which exhibit clear grains corresponding to the (2 2 2), (4 0 0), (2 2 1) and (4 4 0) planes of the cubic bixbyite In_2_O_3_ lattice with an interplanar distance of 2.93 Å, 2.53 Å, 4.11 Å and 1.78 Å, respectively.

Based on the analysis of our experimental data and the information obtained from the literature, different mechanisms for the effect of hydrogen on the film are proposed to dominate in three temperature zones. When annealed at a low temperature (300–500 °C), H is incorporated into the film and exists in the forms of interstitials (H_i_) and substitutions (H_o_) at the oxygen sites. H_i_ and H_o_ have been reported to act as shallow donors in several oxide semiconductors, including In_2_O_3_ and SnO_2_ [22,23]. However, it has been reported that H_i_ in SnO_2_ is thermally unstable at near room temperature and tends to decay upon annealing, while H_o_ in SnO_2_ is more thermally stable up to ≈500 °C [46]. Therefore, it is predicted that the carrier concentration would increase due to the introduction of H_o_ defects by annealing in hydrogen-containing atmosphere. In this temperature range, some donor defects can also be introduced by removing oxygen atoms that could be released in the form of water molecules after combination with hydrogen. However, this seems not to be the dominant mechanism as the variation of oxygen vacancy concentration is small. In addition, the release of the adsorbed water after annealing, as indicated by the FTIR results, may also contribute to the decrease of film resistivity. The film thickness was slightly reduced due to the creation of oxygen vacancies and the release of the adsorbed water after annealing. When annealed at an intermediate temperature (500–700 °C), the H_o_ defects are thermally unstable and expected to decay with annealing temperature. Meanwhile, some metallic clusters are formed locally in the film (observed by XRD and TEM) due to the reduction of the oxide film by H_2_ as expressed by the following reactions:

(2) $${\mathrm{In}}_{2}O_{3-x}+H\to \mathrm{In}+H_{2}O\;\left(g\right)$$

(3) $${\mathrm{SnO}}_{2-x}+H\to \mathrm{Sn}+H_{2}O\;\left(g\right)$$

where g denotes the gaseous phase. In this temperature zone, the film thickness modestly decreases due to the lattice contraction as many vacancy defects exist in the film and the partial reduction of the oxide. The decrease in oxygen vacancy concentration is possibly due to the redistribution of oxygen atoms between the metallic cluster region and the oxide lattice region. Namely, the oxygen vacancies in the oxide lattice region may be passivated by the oxygen atoms released from the metallic cluster region. However, this seems not to be the main reason for the decrease in carrier concentration as the variation of oxygen vacancy concentration is small. When annealed at a high temperature (700–800 °C), the metal oxide film was severely reduced to metallic particles and further reacted with hydrogen, releasing gaseous hydride as expressed by the following reactions [47,48]:

(4) $$\mathrm{In}+H\to {\mathrm{InH}}_{3}\;\left(g\right)$$

(5) $$\mathrm{Sn}+H\to {\mathrm{SnH}}_{4}\;\left(g\right)$$

In this temperature zone, the film thickness would be dramatically decreased until it totally vanished after being annealed at 800 °C. 

**Figure 7:**
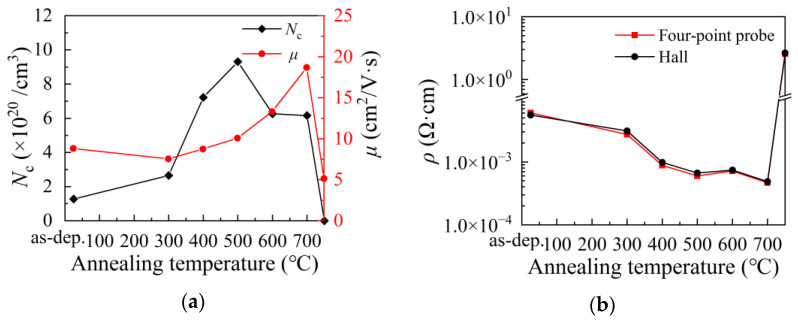
The variation of (**a**) carrier concentration (*N*_c_), mobility (*μ*) and (**b**) resistivity (*ρ*) of the film with annealing temperature.

Figure 7a shows the variation of carrier concentration and mobility of the film with annealing temperature. The carrier concentration increases with annealing temperature in the range of 300–500 °C before it decreases with annealing temperature in the range of 500–750 °C. Assume that the carrier concentration only depended on the oxygen vacancy concentration with a directly proportional relationship, the increments of carrier concentration would be expected to be 2.8 × 10^20^ cm^−^^3^ and 1.4 × 10^20^ cm^−^^3^ after annealed at 500 °C taking the variations of carrier concentration with working pressure (Δ*N*_c_ = 1.2 × 10^20^ cm^−^^3^) and average power (Δ*N*_c_ = 6.0 × 10^19^ cm^−^^3^) as references, respectively. Actually, a much large increment (Δ*N*_c_ = 8.0 × 10^20^ cm^−^^3^) was observed. Therefore, the variation of carrier concentration should be primarily ascribed to the creation and decay of the H_o_ dopant, although the evolution of carrier concentration is approximately the same as the evolution of the oxygen vacancy concentration reported in Table 3. The H_o_ dopant in ITO film is thermally stable and active in contributing free electrons to the conduction band at a low temperature of 300–500 °C. However, the H_o_ dopants are thermally unstable at higher temperatures (>500 °C) and tend to decay with increasing annealing temperature, leading to a decrease in carrier concentration. The mobility of free electrons slightly decreases after annealed at 300 °C, possibly due to the amorphous-like structure of the film surface. Further, increasing the annealing temperature to 400–700 °C leads to an increase in mobility, which should be ascribed to the increase in grain size and the reduction of grain boundary scattering. However, The electron mobility sharply decreases after annealed at 750 °C. On the one hand, the sharp decrease in mobility should be ascribed to the severe surface scattering as the film thickness is greatly reduced to 20.6 nm after annealed. On the other hand, the scattering by increased density of point defects and metallic inclusion should play a role in decreasing the electron mobility in the film. As a result, the film resistivity (*ρ*) shown in Figure 7b decreases with annealing temperature at 300–700 °C, then dramatically increases after being annealed at 750 °C.

**Figure 8:**
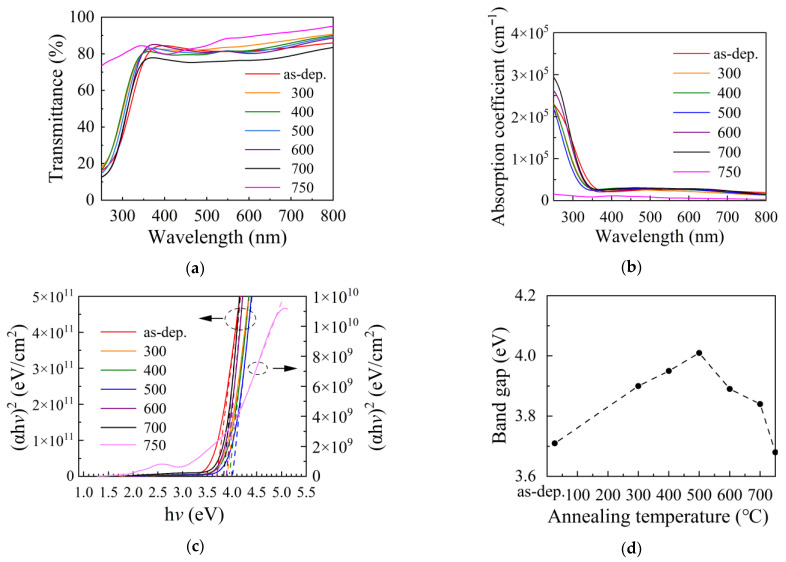
(**a**) The transmittance spectra, (**b**) light absorption coefficient (α) spectra and (**c**) Tauc’s plots for the ITO films annealed at different temperatures. (**d**) The variation of optical band gap with annealing temperature.

Figure 8a shows the transmittance (*T*) spectra for the ITO films annealed at different temperatures. The films all exhibit a similar high transmittance of >80% in the visible light range (350–800 nm). The film transmittance sharply decreases at the ultraviolet light range (300–350 nm) for the as-deposited film, and the films annealed at 300–700 °C due to the band-to-band transition. The film annealed at 750 °C is highly transparent due to the very small thickness (28 nm). The absorbance (α) spectra plotted in Figure 8b were calculated using the following expression [49]:

(6) $$\alpha =\frac{1}{d}ln\left(\frac{1}{T}\right)z$$

The absorbance is very weak in the visible light range (350–800 °C) for all films and sharply increases in the shorter light range due to band-to-band transition. The extremely weak absorption of the ITO film annealed at 750 °C should be due to the small film thickness. The absorption edge in the transmittance and absorbance spectra exhibit a blue shift after annealing treatment, indicating that the optical band gap changed after annealing. The optical band gap was obtained from the Tauc plot in Figure 8c according to the following formula [50]:

(7) $${\left(\alpha hv\right)}^{2}=A\left(hv-E_{g}\right)$$

where h*v* is the energy of incident photons, A is a constant, and *E*_g_ is the optical band gap. Figure 8d shows the variation of the optical band gap with annealing temperature. The optical band gap increases with annealing temperature at 300–500 °C. Then, it decreases with annealing temperature at 500–750 °C. This developing trend seems to be positively related to that of free carrier concentration, suggesting that the Moss–Burstein effect, which often arises in heavily doped semiconductors and manifests as widening of optical band gap by increasing carrier concentration, plays an important role in influencing the optical band gap. The existence of metallic clusters does not lead to observable deterioration of the optical properties of the film, although deterioration has been observed in ITO treated by H_2_ plasma and annealed in an H_2_-containing atmosphere reported by other groups [18,20].

Table 5 lists the main results in this study and previous results for HiPIMS-ITO films and ITO films prepared by other techniques. The annealing processes in the table were all carried out in hydrogen-containing ambient (with different H_2_/N_2_ ratios). For the as-deposited films prepared by DC magnetron sputtering (DCMS) and RF magnetron sputtering (RFMS), the resistivity is (3.4–6.2) × 10^−4^ Ω·cm and the average transmittance in the visible light range (Ave. T) is 70–90% [21,51,52]. After annealed at 500 °C, the resistivity decreases to (2.2–4.1) × 10^−4^ Ω·cm and the Ave. T is improved to 86–90%. For the ITO films prepared by sol-gel method and annealed at 600 °C, the resistivity is 4.4 × 10^−2^ Ω·cm and the Ave. T is 86% [53]. For the ITO films prepared by e-beam evaporation, the resistivity is 4.4 × 10^−2^ Ω·cm and the Ave. T is 32% [54]. These values are significantly improved to 5.8 × 10^−4^ Ω·cm and 61% after annealed at 200 °C. For the as-deposited HiPIMS-ITO films, the resistivity is (4.0–6.0) × 10^−3^ Ω·cm and the Ave. T is 78–82% [16,55,56]. The resistivity decreases to 6.7 × 10^−4^ Ω·cm and the Ave. T is almost unchanged after being annealed at 500 °C. Overall speaking, the ITO films prepared by DCMS and RFMS have the best performance in terms of the electrical and optical properties both at the as-deposited and annealed states; the ITO films prepared by HiPIMS score the secondary level at the as-deposited state but achieves similar level to the DCMS and RFMS after annealed; the ITO films prepared by e-beam evaporation have inferior performance at the as-deposited state, but the resistivity can be improved to the similar level of the DCMS and RFMS while the transmittance remains poor after annealed; the ITO films prepared by the sol-gel method have inferior performance even after annealing. The results of this work fill in the blank of the annealing data for the HiPIMS-ITO film. 

## 4. Conclusions

ITO films were prepared by an in-line HiPIMS system. Annealing treatment in hydrogen-containing forming gas can effectively reduce the film resistivity without observable deterioration of the optical properties. The lowest film resistivity of 6.7 × 10^−4^ Ω·cm was obtained after annealed at 700 °C for 40 min. The hydrogen (H_2_) plays different roles in influencing the film properties at different annealing temperatures: (1) hydrogen atoms substitute the oxygen atoms (H_o_), acting as shallow donors that contribute to the increase of carrier concentration at a low annealing temperature of 300–500 °C; (2) the substitutional hydrogen (H_o_) is thermally unstable at an intermediate annealing temperature of 500–700 °C and decays with increasing annealing temperature, leading to the decrease of carrier concentration; In addition, metallic clusters are formed by the reduction of H_2_; (3) the hydrogen gas reduces the metal oxide severely, producing volatile metal hydride, leading to a dramatic decrease of the film thickness and finally vanish of the film at a high annealing temperature of 700–800 °C. The substitutional doping of H and the modest reduction by H_2_ both lead to the increase of free carrier concentration in the film and the decrease of film resistivity.

## Figures and Tables

**Table 1:** Deposition parameters for the high-power impulse magnetron sputtering (HIPIMS) process.

Parameter	Value
Base pressure (×10^−^^5^ Pa)	6.7
Working pressure (Pa)	8.0
Distance of substrate-to-target (mm)	52
Average power (W)	500
Flow rate of Ar (sccm)	40
Deposition temperature (°C)	25
Frequency (Hz)	1000
Pulse length (μs)	100
Duty cycle (%)	10

**Table 2:** Parameters for the annealing process of ITO film.

Parameter	Value
Temperature (°C)	300–800
Duration (min)	40
Atmosphere	N_2_ (95%) + H_2_ (5%)
Gas flow rate (L/min)	1

**Table 3:** The Sn doping efficiency and oxygen vacancy concentration at different annealing temperatures.

Temperature (°C)	Sn^4+^/(Sn^4+^ + Sn^2+^) (%)	O_V_/(O_V_ + O_L_) (%)
25	75.0	45.1
300	73.0	45.6
400	70.2	47.2
500	69.3	47.6
600	70.0	46.5
700	71.9	44.1
750	69.8	42.0

**Table 4:** Assignments of the infrared absorption peaks of ITO film.

Wavenumber (cm^−1^)	Assignments
3680	Si-OH
2260, 2020	Si-H
1904, 1802	C-O
1603, 1460	water
1350,832	C-H
1291, 531	C-O
520	In-O

**Table 5:** Comparison of the results of this study with previous results.

Method	H_2_/N_2_ Ratio (%)	Annealing Temp. (°C)	ρ (Ω·cm)		Ave. T (%)		Ref.
			As-dep.	Post-ann.	As-dep.	Post-ann.	
DCMS	50	500	3.4 × 10^−4^	2.2 × 10^−4^	88	90	[21]
DCMS	100	500	6.2 × 10^−4^	2.7 × 10^−4^	90	92	[51]
RFMS	2	500	6.0 × 10^−4^	4.1 × 10^−4^	70	86	[52]
Sol-gel	3.75	600	N.A.	4.4 × 10^−2^	N.A.	86	[53]
E-beam evaporation	20	200	5.6 × 10^−2^	5.8 × 10^−4^	32	61	[54]
HiPIMS	N.A.	N.A.	4.0 × 10^−3^	N.A.	N.A.	N.A.	[16]
HiPIMS	N.A.	N.A.	4.0 × 10^−3^	N.A.	N.A.	N.A.	[55]
HiPIMS	N.A.	N.A.	6.0 × 10^−3^	N.A.	82	N.A.	[56]
HiPIMS	5	500	5.6 × 10^−3^	6.7 × 10^−4^	78	78	This work

Notes: N.A. is short for not available; Ave. T is short for average transmittance in the visible light range; As-dep. is short for as-deposited; Post-ann. is short for post-annealed.

## Data Availability

Not applicable.
